# Seroprevalence of hepatitis B virus and hepatitis C virus infection among pregnant women attending antenatal care at Guhala Primary Hospital, Northwestern Ethiopia

**DOI:** 10.1186/s12884-024-06714-6

**Published:** 2024-07-29

**Authors:** Debaka Belete, Engidayehu Fekadie, Melkamu Kassaw, Melaku Fenta, Azanu Jegnie, Tigist Mulu, Gashaw Adane, Wondwossen Abebe, Azanaw Amare

**Affiliations:** 1 Department of Medical Microbiology, College of Medicine and Health Sciences, University of Gondar, Gondar, Ethiopia; 2 School of Biomedical and Laboratory Sciences, College of Medicine and Health Sciences, University of Gondar, Gondar, Ethiopia; 3 Department of Immunology and Molecular Biology, College of Medicine and Health Sciences, University of Gondar, Gondar, Ethiopia

**Keywords:** Hepatitis B virus, Hepatitis C virus, Prevalence, Pregnant women, Guhala, Ethiopia

## Abstract

**Background:**

Hepatitis B virus (HBV) and Hepatitis C Virus (HCV) infections are global issues that disproportionately affect developing countries. Pregnancy-related HBV and HCV infections are associated with a high risk of vertical transmission and complications for the mother as well as the newborn. Therefore, this study aims to determine the seroprevalence of hepatitis B virus and hepatitis C virus infection among pregnant women attending antenatal care at Guhala Primary Hospital, Northwestern Ethiopia.

**Methods:**

A hospital-based retrospective study was conducted from July to September 2022 on HBV and HCV registered books from September 1, 2017, to August 30, 2019, for a year. The presence of HBsAg and anti-HCV in serum was detected using the One Step Cassette Style HBsAg and anti-HCV antibody test kit. Data were analyzed using SPSS version 26 software.

**Results:**

In this study, a total of 2252 participants for HBsAg and 538 participants for ant-HCV rapid tests of records in the laboratory logbook were included. The mean age of the study participants was 25.6years (± 5.8SD). The overall prevalence of HBsAg and anti-HCV was 6.0% (134/2252) and 2.4% (13/538), respectively. There were 0.4% (2/538) coinfection results between HBV and HCV among pregnant women.

**Conclusion and recommendation:**

In this study, intermediate seroprevalence of HBV and HCV infection was detected among pregnant women attending antenatal care. The Hepatitis B virus was predominantly higher among pregnant women aged between 25 and 34 years. To manage and stop the potential vertical transmission of these viral agents during the early stages of pregnancy, routine prenatal testing for HBV and HCV infections should be taken into consideration.

## Introduction

Hepatitis B virus (HBV) and Hepatitis C virus (HCV) infection is an emerging worldwide public health concern affecting millions of people each year [1–3]. HBV and HCV are the etiology of HBV and HCV infection. These infections can cause both acute and chronic hepatitis, as well as possibly induce cirrhosis, liver cancer, or even death in those who are affected [3–5].

Globally, HBV and HCV cause chronic infections in 254 million and 50 million persons worldwide, respectively, and these diseases result in 1.1 million and 242, 000 deaths annually, respectively [6]. In developing countries like Asia and Africa, HBV and HCV infections are very common [7]. The World Health Organization reported that there were 1.2 million and 1.0 new cases of chronic HBV and HCV infection worldwide in 2022, respectively [6]. According to WHO estimates between 3 and 4 million people are acquired the disease annually, with the majority of cases occurring in Africa [8]. Other evidence also showed that an estimated 71.1 million people are infected with HCV worldwide [3, 9]. Despite being highly contagious, HBV and HCV are still underdiagnosed and underreported in the majority of African countries [10].

Both HBV and HCV have the potential to be widespread and impact a variety of populations, such as those with HIV, healthcare professionals, the general population of blood donors, pregnant mothers, and their children [11–15]. Numerous studies show that the prevalence of HBV and HCV in pregnant women is now seriously associated with public health [14, 16, 17]. Mother-to-child transmission of HBV, which might be through intrauterine transmission is a common event and causes chronic infection of the virus [18]. The seroprevalence of HBV among pregnant women in Ethiopia ranges from 4.5 to 7.9%, which is indicative of an intermediate degree of endemicity for the virus [19–23]. The seroprevalence of HCV, which ranges from 0.26 to 8.07%, was shown to be lower than that of HBV in the majority of Ethiopian studies [17, 23–25].

Childbearing women can potentially transmit HBV and HCV to their children’s. They transmit an infection to newborns usually during birth or soon after birth following close contact. There is a higher likelihood of vertical transmission of infection from mothers to offspring in 4.6% and 1.6% of babies delivered to pregnant women with HBV and HCV infections, respectively [26]. Newborns who are exposed to HBV will have almost 85–90% risk of developing chronic liver diseases [27].

Preterm delivery, placental separation, vaginal bleeding, early rupture of the membranes, and mortality are among the many issues that can arise from maternal infection with HBV and HCV during pregnancy [28, 29]. A significant risk of neonatal hepatitis is also linked to it, and this can result in liver cirrhosis and hepatocellular cancer in young adulthood [30, 31]. Generally, in the health system of Ethiopia, the health burden due to viral hepatitis is still less [32, 33]. Even though viral hepatitis screening is advised during routine antenatal care (ANC), routine antenatal screening of pregnant women is not common and obligatory in Ethiopia [34]. Recent research showed that both the general population and healthcare professionals reported having little knowledge of the hepatitis virus [25, 32]. Therefore, this study aimed to determine the seroprevalence of HBV and HCV among pregnant women for the last three years using a laboratory-recorded book to fill the current epidemiologic gap in the area.

## Materials and methods

### Study design, area, and period

A hospital-based retrospective study was conducted among pregnant women from HBV and HCV registered books at Guhala Primary Hospital, Northwestern Ethiopia, from September 1, 2017, to August 30, 2019. Guhala is the capital town of East Belesa, located northwest of Addis Ababa (the capital city of Ethiopia), which is 718 km away. Guhala town has an estimated total population of 148,758, of whom 75,732 were males and 73,026 were females. The town has only one public primary hospital, one health center, one health post, and four private health facilities. Guhala Primary Hospital is the only public hospital. Today, the hospital is providing health services to a total population of about 148,758. The data collection period was from July to September 2022.

### Source population, study population, sample size, and sampling technique

The source populations were all pregnant mothers who were attending ANC in Guhala Primary Hospital in northwestern Ethiopia. The study population consisted of all pregnant mothers who had requested Hepatitis B surface Antigen (HBsAg) and anti-HCV screening from the respective ANC follow-up and gave blood for HBsAg and anti-HCV screening and recorded in the Laboratory logbook at the Guhala Primary Hospital from September 1, 2017, to August 30, 2019. The record of data HBsAg and anti-HCV statuses, years, and ages were included in this study. Patient information with incomplete demographic characteristics, and test results in the log book was excluded.

### Data collection tools

We collected data from all pregnant women who screened for HBsAg and anti-HCV from serology registration logbooks using a structured checklist. Client information, including age, laboratory results, and year of diagnosis was obtained from the serology laboratory registration logbooks.

### Laboratory methods

The hepatitis B surface antigen (HBsAg) and anti-HCV antibody test were requested as part of the antenatal care panel. A 5 ml of venous blood was collected from each pregnant woman. Then, the serum was separated by centrifugation at 5000 rpm for 15 min and tested for the presence of HBsAg and ant-HCV antibodies using a one-step HBsAg test strip (Nantong Diagnosis Technology Co., Ltd., China) and a one-step HCV test strip (Nantong Diagnosis Technology Co., Ltd., China), respectively, by following the instructions of the manufacturer. The sensitivity and specificity of rapid test kits of HBsAg and one-step HCV test strips were 99.1% and 99.6%, respectively.

### Data quality control

We used different methods to ensure that the collected data had the required quality. For instance, we used a structured data collection checklist and regular communication with the hospital staff working at the serology laboratory. Finally, we checked the collected data, cleaned it manually, and entered SPSS version 26 for analysis.

### Data processing and analysis

All participants’ information and laboratory data were entered and then analyzed using SPSS version 26 (IBM Corp., Armonk, NY). Descriptive analysis was used to describe and calculate the frequencies and percentages of variables.

## Results

### Socio-demographic characteristics of study participants

Over the five years (2017–2019), a total of 2,252 serologically tested pregnant women were included. The age of patients’ ranges from fifteen to sixty-five years and the mean age of the patients were 25.6 years (± 5.8 SD). Most of the samples were tested in 2019 (37.3%), followed by 2018 (33.5%), and 2017 (29.3%). Most of the samples came from pregnant women between the ages of 15 and 24 years (45.6%), followed by those the age between of 25–34 (44.2%) and those the ages of 35–44 years (9.6%). On the other hand, over the five years (2017–2019), a total of 538 samples collected from pregnant women were tested for anti-HCV at the Guhala Primary Hospital serology laboratory. Most of them, 45.3% (243) were between the ages of 15 and 24, followed by those the age between of 25–34 (44.3%) (Table 1).

**Table 1:** Socio-demographic characteristics of study participants in Guhala Primary Hospital, Northwestern, Ethiopia, from September 1, 2017 to August 30, 2019

Variable	Category	HBsAg suspected participants (*N* = 2252)	anti-HCV suspected participants (*N* = 538)
		Frequency n(%)	Frequency n(%)
Age in year	15–24	1028 (45.6%)	243 (45.3%)
	25–34	995 (44.2%)	238 (44.3%)
	35–44	216 (9.6%)	51 (9.5%)
	> 45	13 (0.6%)	2 (0.4%)
Year of diagnoses	2017	659 (29.3%)	18 (3.4%)
	2018	839 (37.3%)	263 (48.9%)
	2019	754 (33.5%)	257 (47.7%)

### Sero-prevalence of HBV and HCV among pregnant women

The overall prevalence of HBsAg and anti-HCV were 6% (134/2252) (95% CI = 5–7%) and 2.4% (13/538) (95% CI = 1.3–3.7%), respectively. Hepatitis B virus was predominantly higher among pregnant women aged between 25 and 34 years old 7.13% (71/995) followed by individuals aged between 15 and 24 years old 5.0% (51/1028). On the other hand, the prevalence of hepatitis C virus was high frequency among pregnant women in the age group between 35 and 44 years old 6% (3/51). There were 0.4% (2/538) coinfection results between HBV and HCV among pregnant women. Moreover, a high percentage of HBsAg and anti-HCV were seen in the year 2018 and 2019, respectively (Table 2).

**Table 2:** Sero-prevalence of HBV and HCV among pregnant women in Guhala Primary Hospital Northwestern Ethiopia, from September 1, 2017 to August 30, 2019

Variables	HBsAg suspected *N* (%)	HBV positive *N* (%)	HCV suspected *N* (%)	anti-HCV positive *N* (%)	HBsAg and HCV positive *N* (%)	
Age						
15–24	1028(45.6%)	51(5.0%)	243 (45.3%)	5 (2.1%)	0	
25–34	995(44.2%)	71 (7.1%)	238 (44.3%)	4 (1.7%)	1(0.2%)	
35–44	216(9.6%)	11(5.1%)	51 (9.5%)	3 (6.0%)	1(0.2%)	
>45	13(0.6%)	1 (7.7%)	2 (0.4%)	1 (50%)	0	
Year						
2017	659(29.3%)	31 (4.7%)	18 (3.4%)	1 (5.5%)	0	
2018	839(37.2)	60 (7.2%)	263 (48.9%)	4 (1.5%)	1(0.2%)	
2019	754(33.5)	43 (5.7%)	257 (47.7%)	8 (3.1%)	1(0.2%)	

## Discussion

The seroprevalence of HBsAg in the current study was 6% (134/2252) 95% CI = 5–7%), and this infectivity rate can be considered as a highly endemic scenario for the virus [35]. The seroprevalence of HBsAg in the present study was comparable with other similar studies conducted in West Hararghe, Ethiopia (6.1%) [36], Borena Zone (5.7%) [37], Nekemte (5.8%) [38], Bishoftu (6.5%) [39], and Dilla, Ethiopia (5.1%). However this report is lower than the study conducted in Debre Markos, Ethiopia (8.3%) [40], Borumeda, Ethiopia (8.1%) [41], Adigrat General Hospital, Ethiopia (9.2%) [42] Jijiga, Ethiopia 8.5% [43], Southern, Ethiopia (10.9%) [44] and (7.8%) [45], Gedeo zone (9.2%) [46], Gambella (7.9%) [22], the Republic of South Sudan (11%) [47] and (8.5%) [48], Tanzania, (8.03%) [49], Uganda (11.8%), Gambia (9.2%) [50], Ghana (7.7%) [51], Cameroon (10.78%) [52], and Nigerian (14.1%) [53].

On the contrary, the result of this study was relatively high when compared to the result reported in Gondar, Ethiopia (4.6%) [54], Felegehiwot referral hospitals, Ethiopia (4.7%) [55], Bahir Dar, Ethiopia (3.9%) [13] and (3.8%) [56], Gandhi memorial hospital, Ethiopia (2.3%) [57], Addis Ababa, Ethiopia (4.5%) [58], Shone (4.9%) [59], Agena health center in the Gurage zone (4.1%) [60], Ambo town, Ethiopia (4.99%) [61], Southern, Ethiopia (4.5%) [23], Rwanda (3.1%) [62], Uganda (2.1%) [63], and Pakistan (3.7%) [64]. The variation may result from differences in the study period, sample size, population demographics, and clinical conditions of study participants across different locations, and cultural practices and methods utilized for infection prevention and unsafe behavior practices. When compared to other research conducted outside of the African continent, the study’s findings were higher than the study conducted in Pakistan (2.78%) [65], Turkey (2.1%) [66], and India (1.6%) [15]. The variation may be the result of the routine immunization and screening programs in those countries.

In the present study, we found that the majority (7.1%) of the study participants infected by hepatitis B were in the age group 25–34 years. This finding is similar to a study in Adigrat, Ethiopia where the majority of the study participants infected by hepatitis B were in the age group 25–34 years [42].

In this study, the seroprevalence of HBV among pregnant mothers was higher than that of HCV (6% versus 2.4%). This is similar to studies conducted in Ethiopia (8.1% versus 3.2%) [41], ( 4.6% versus 1.6%) [54], and Pakistan (3.7% versus 2.1%) [64]. In contrast to this a study conducted in West Oromia, Ethiopia, showed a higher HCV rate (8.07%) than HBV rate (2.4%) among pregnant women [17]. Another similar study in the Democratic Republic of Congo also showed a slightly higher HCV rate of infectivity (4.8%) than HBV (3.9%) [67]. In Egypt, HCV was found to be the more common infection than HBV, despite the extremely low prevalence [14]. This dominance of HCV over HBV in other studies might be associated with a non-detection ability of HBsAg due to mutation on the surface antigen gene [68].

In the present study, we found 2.4% (95% CI = 1.3–3.7%) of the overall seroprevalence of HCV Among pregnant women screened for HCV infection in Guhala Primary Hospital. This finding is consistent with the study conducted in Gondar Ethiopia (1.6%) [54], southwest, Ethiopia (2.3%) [69] Southern Ethiopia 1.83% [23], and systematic review and meta-analysis in Ethiopia (1.83%) [24]. However, this finding is lower than the study conducted in the East Wollega zone (8.07%) [17] and Ghana (7.7%) [70]. On the other hand, our study is higher than the study conducted in Bahir Dar, Ethiopia, (0.62%) [71] and (0.26%) [25], and Dessie, Ethiopia (0.7%) [72].

In the present study, there was a slight connectivity rate between the two viruses among pregnant women. This study is concordant with the study conducted in Ethiopia [54], Nigeria [73], and Ghana [70], where, a similar scenario in which the presence of HBV and HCV connectivity was documented. Besides, this study is discordant with the study conducted in Ethiopia [25, 41], Nigeria [74, 75], and Pakistan [64], Where, the absence of HBV and HCV connectivity was documented. A study carried out in China clarified HCV spontaneous clearance among HBV/HCV-co-infected study subjects, and it also demonstrated that female gender, concentration of HBV DNA, and genotype are associated with enhanced spontaneous clearance of HCV [76].

### Limitations of the study

The study has drawbacks related to missing data and an insufficient document-keeping system since it relied on secondary sources. Another drawback is that only the detection of HBsAg and anti-HCV has been possible in laboratory testing and enzyme linked immunosorbant assay and the Nucleic Acid Amplification Technique was not employed.

## Conclusions and recommendation

In the current study, the seroprevalence of the two common viral hepatitis infections was intermediate, and the rate of HBV and HCV particularly can be considered in the intermediate endemic category of the WHO classification scheme. Collaboration amongst various stakeholders is essential to reducing the prevalence of infections among pregnant women. It is recommended that pregnant women undergo regular screening, that women of childbearing age receive the Hepatitis B vaccine, and that health education programs raising awareness of HBV and HCV should take place. Furthermore, we recommend studies be conducted to compare the real infections of HBV and HCV by using more advanced laboratory techniques among pregnant women living in rural and urban areas of Ethiopia.

## Data Availability

The datasets generated and/or analysed during the current study are not publicly available due ongoing analyssi but are available from the corresponding author on reasonable request.

## Abbreviations

Anti-HCV: Anti Hepatitis C virus Antibody
ANC: Antenatal Care
HBsAg: Hepatitis B surface antigen
HBV: Hepatitis B virus
HCV: Hepatitis C virus
WHO: World Health Organization
